# A porphyrin-centred fullerene tetramer containing an N@C_60_ substituent

**DOI:** 10.1098/rsos.180338

**Published:** 2018-07-18

**Authors:** Harry Macpherson, Stuart Cornes, Shen Zhou, Kyriakos Porfyrakis

**Affiliations:** Department of Materials, Oxford University, Oxford OX1 3PH, UK

**Keywords:** fullerenes, nanomaterials, quantum information processing

## Abstract

An N@C_60_-containing C_60_ tetramer was synthesized by quadruple 1,3-dipolar cycloaddition (Prato) reaction. This molecule demonstrates the N@C_60_ qubit's ability to form covalently linked arrays. Furthermore, it provides a promising scaffold with which to measure multiple qubit–qubit interactions; which must be well characterized for a functioning quantum information processing architecture.

## 1. Introduction

N@C_60_, single atomic nitrogen incarcerated within a C_60_ fullerene [1], has been proposed as a qubit architecture [2,3] due to the remarkably long relaxation times of its p-electron spins (*T*_1_ = 0.375 ms, *T*_2_ = 0.25 ms) [4]. Versatile methodologies for the chemical functionalization of N@C_60_ [5] and large-scale production (approx. 10^15^ qubits per hour) mark it as a promising alternative to today's predominantly inorganic QIP systems. For the N@C_60_ system to be realized as a viable QIP system, qubit–qubit interactions must be well characterized, controlled entanglement must be demonstrated and scalable arrays must be assembled [6]. Qubit–qubit interactions were previously characterized by the measurement of the paramagnetic dipolar coupling strength between two units in an N@C_60_ dimer by continuous wave electron paramagnetic resonance (cw-EPR) spectroscopy [7]. However, the interactions of arrays of more than two qubits are yet to be explored. To this end, herein we describe a tetraphenylporphyrin-centred N@C_60_-containing C_60_ tetramer, which provides a scaffold for the measurement of dipolar interactions in arrays of up to four qubits, and is the largest N@C_60_-containing C_60_ array synthesized to date.

**Figure 1.**
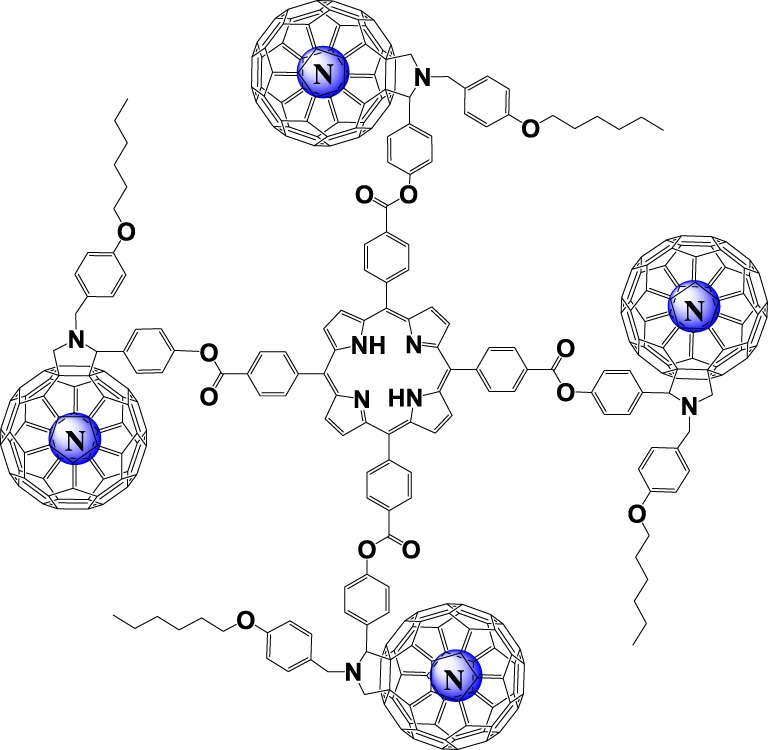
Cartoon showing the tetramer scaffold when fully filled with atomic nitrogen.

N@C_60_ compatible synthesis of this tetramer is the proof of concept that it can be used to characterize many-qubit interactions if the reaction is repeated with high purity N@C_60_ (figure 1).

## 2. Results and discussion

Tetraphenylporphyrin was chosen as the central moiety for the tetramer for its planarity and fourfold symmetry, which would be beneficial for future dipolar coupling studies. Attachment of fullerene to the central porphyrin was undertaken using a Prato cycloaddition reaction due to its proven compatibility with N@C_60_ [7], along with the wide range of amino acid and aldehyde starting materials it is compatible with. The N@C_60_ used for the reaction was produced by ion implantation as previously reported [8] and purified to an N@C_60_ : C_60_ ratio (spin fraction) of 0.15% by high pressure liquid chromatography (HPLC) [9]. Owing to the time-consuming and expensive nature of the ion implantation process and HPLC purification, it was not possible to use a higher spin fraction starting material.

**Scheme 1.**
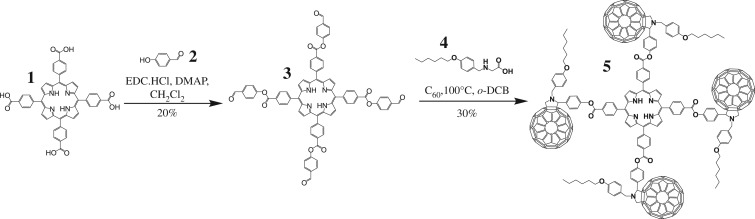
Synthesis of the target tetramer **5**.

Synthesis of the target tetramer is shown in scheme 1. Initially, preparation of a porphyrin-centred tetra-aldehyde **3** was undertaken through reaction of the commercially available tetra-acid **1** with 4-hydroxybenzaldehyde **2** under EDC coupling conditions. Purification via column chromatography (SiO_2_) followed by subsequent recrystallization (CH_2_Cl_2_) afforded **3** as a purple solid in 20% yield. Importantly, this synthetic route was chosen to produce a tetra-aldehyde, instead of the direct reduction of **1** into its corresponding tetra-aldehyde, to minimize steric hindrance in the final fullerene-containing product. A faster Prato reaction and therefore shorter heating time would be expected if N@C_60_/C_60_ was first Prato functionalized with a nucleophilic functional group and then coupled with tetra-acid **1**. However, this approach was precluded by significantly decreased reactivity of nucleophiles in proximity to C_60_ [10].

The target tetramer was, therefore, formed via a quadruple Prato reaction involving tetra-aldehyde **3**. This was initially attempted using pure C_60_, with a mixture of **3**, amino acid **4** and C_60_ heated at 100°C for 90 min in *ortho*-dichlorobenzene (*o-*DCB). Importantly, the secondary amino acid *N*-(4(hexyloxy)benzyl)glycine **4** was used, as it has been shown in previous work to react very quickly under these conditions, and would hence allow for minimal thermal decomposition of N@C_60_ [7,11] to occur in the preparation of the corresponding N@C_60_-containing tetramer. Its accelerated reactivity is rationalized through a *π*−*π* stacking interaction between the aromatic group of the amino acid and the fullerene cage, which possibly stabilizes the ylide intermediate and should increase the probability of a successful collision between the three reagents [7].

Aliquots of the mixture were taken periodically to monitor the reaction progression by matrix-assisted laser desorption/ionization mass spectrometry [12] (see the electronic supplementary material). After 5 min, monomer and dimer were observed, followed by dimer, trimer and tetramer at 15 min. No further changes to the spectrum were observed after 90 min, with only trimer and tetramer peaks observed. Purification using column chromatography (SiO_2_) was then undertaken, with excess C_60_ initially eluted with *o*-DCB after which the tetramer product was eluted with 1% ethyl acetate/*o*-DCB in 30% yield.

**5** was characterized by proton nuclear magnetic resonance (^1^H-NMR) spectroscopy in *d*_4_-*o*-DCB. The room temperature spectrum has broad peaks, which is probably the result of aggregation, which is further indicated by the incredibly poor solubility of **5**. The fact that poor solubility of **3** was also observed would suggest stacking of the porphyrin moiety as a likely cause [13,14]. A spectrum collected at 373 K shows substantially sharper peaks and shows all expected resonances, including porphyrin NH protons at −3 ppm (see the electronic supplementary material). Importantly, the absence of an aldehyde resonance at approximately 10 ppm confirms that no monomer, dimer or trimer impurities are present. Owing to the poor solubility of the tetramer, it did not prove possible to obtain a ^13^C-NMR spectrum.

**Figure 2.**
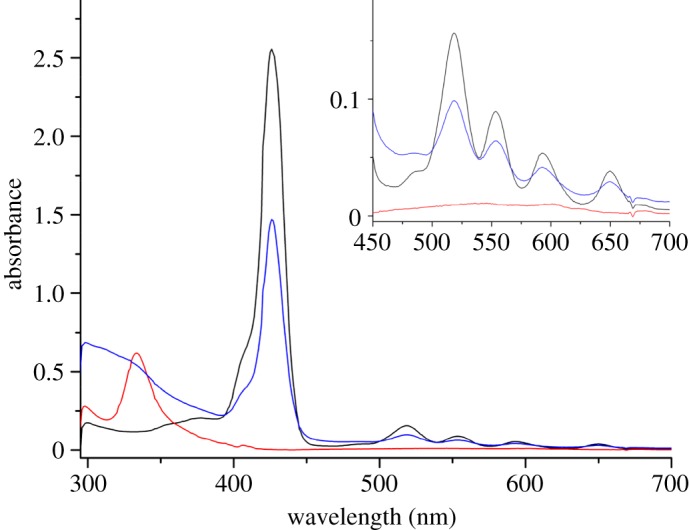
UV–vis spectrum of **3** (black line), **5** (blue line) and C_60_ (red line) in *o*-DCB (approx. 10 µM).

Ultraviolet-visible (UV–vis) spectroscopy was performed on **3** and **5** and on pristine C_60_ in *o-*DCB. Figure 2 shows the characteristic porphyrin Soret band at around 425 nm is present in both **3** and **5**, with the spectrum of **5** being dominated by the porphyrin absorptions. This is in agreement with two previously studied porphyrin-based C_60_ tetramers [15,16]. The peak typically observed at 420 nm [17], which would confirm functionalization of C_60_ to a fulleropyrrolidine, is presumably obscured by this broad absorption band; however, functionalization of the porphyrin is indicated by an additional peak at around 325 nm.

**Figure 3.**
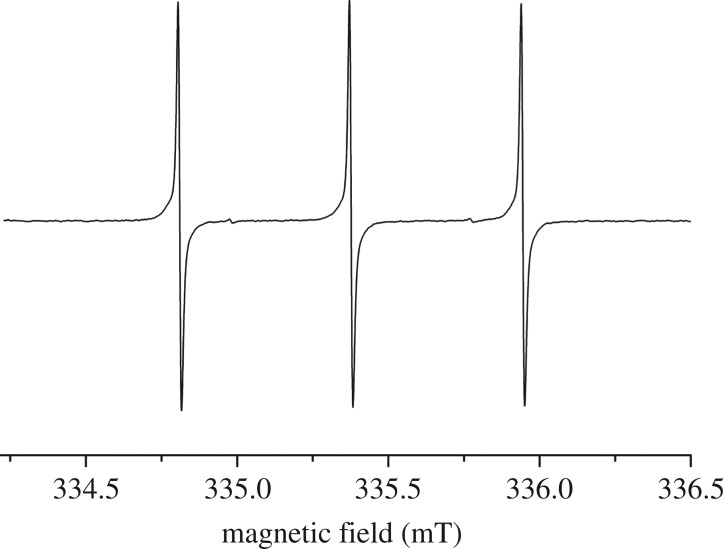
X-band cw-EPR spectrum of **5** in *o*-DCB taken at 298 K.

Synthesis of the corresponding N@C_60_ containing tetramer **5** was then achieved using the same reaction conditions, with a 60-min reaction time used to minimize thermal decomposition of N@C_60_. In order to confirm the presence of endohedral nitrogen in **5**, a room temperature cw-EPR spectrum was acquired in *o-*DCB (figure 3). Three sharp peaks can be seen, corresponding to each ^14^N nuclear spin projection (*M*_I_ = −1, *M*_I_ = 0 and *M*_I_ = +1) [4] and two smaller peaks in between, corresponding to a small, naturally occurring ^15^N (*M*_I_ = ±1/2) impurity, which generally is only visible in high spin concentration samples.

**Figure 4.**
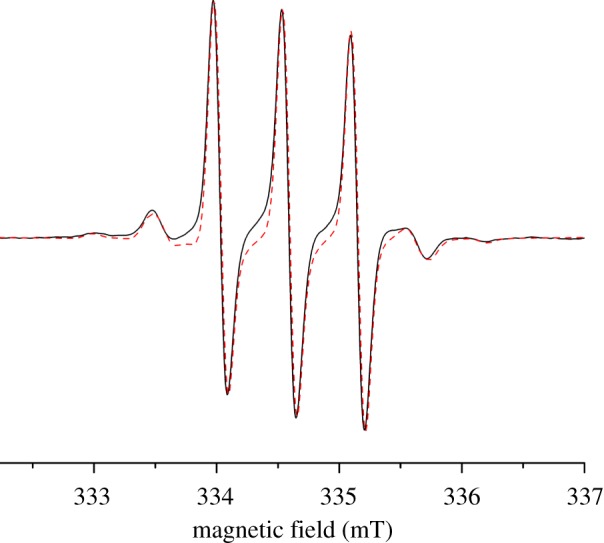
X-band cw-EPR spectrum of **5** in *o*-DCB at 100 K (black line); Easyspin simulation (red dashed line).

The solution was then frozen in liquid nitrogen and a solid-state cw-EPR spectrum was collected (figure 4). Four visible zero field splitting (ZFS) peaks indicate breakdown of the symmetry of atomic nitrogen's electronic environment and therefore confirm functionalization of N@C_60_ [18]. Unfunctionalized C_60_ has icosahedral symmetry [19] and therefore nitrogen's three-spin transitions are degenerate. Functionalization of the cage breaks the p-orbital symmetry and lifts the degeneracy of the three-spin transitions, giving rise to ZFS peaks [20]. The ZFS tensor is traceless and therefore its effect on the EPR signal is averaged out by the rapid tumbling of the molecules in liquid solution and so this effect can only be seen in frozen solution [20]. Solid solution strain or g-strain acts to broaden the primary peaks [21]. An electron gyromagnetic ratio of *g* = 2.00514 and ZFS parameters *D* = 17.71 MHz and *E* = 0.63 MHz were calculated using the EasySpin line fitting tool and are in line with previous Prato functionalized N@C_60_ derivatives [18,22].

Owing to the low spin fraction of the N@C_60_/C_60_ sample used, approximately 99.4% of molecules of tetramer **5** contain no N@C_60_ units, approximately 0.6% contain one N@C_60_, approximately 0.0013% contain two spins, while there are only negligible proportions of molecules containing three and four spins, as calculated using the binomial probability formula. As the concentration of single-spin molecules is over 400 times larger than that of two-spin molecules, the cw-EPR spectra are representative of isolated single-spin molecules. In any reaction to form a covalent array using less than 100% spin fraction starting material, a binomial distribution of products containing different numbers of N@C_60_ units will be produced.

**Figure 5.**
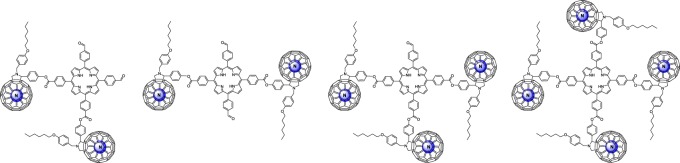
Four spin arrays in which the dipolar coupling strength between adjacent qubits can be evaluated using the tetramer scaffold.

As mentioned previously, the dipolar coupling strength between two N@C_60_ units has been measured in an N@C_60_ dimer, using starting material with a spin fraction greater than 50% [7]. The two-spin signal was found by subtracting a one-spin signal generated by an earlier low spin fraction reaction. By a similar method, four distinct spin interactions that can be hosted in tetramer **5** can be individually characterized (figure 5). An added advantage of tetramer **5** is that this can, in principal, be achieved using only one high spin fraction reaction in the following manner: an aliquot taken after 5 min will contain monomer and two dimers of different configurations and an aliquot taken at 15 min is known to contain trimer, while the final reaction mixture contains tetramer. By isolating each species by chromatography, a one-spin cw-EPR spectrum (the same as that gathered in this study) can be measured from the isolated monomer. The one-spin signal can then be subtracted from the EPR signals of each isolated dimer configuration to give the two-spin signal for each. Each two-spin signal can then be analysed using the EasySpin line fitting tool to determine the dipolar coupling strength for two average spin-to-spin distances, because in each dimer configuration the neighbouring spins are different average distances apart. Likewise, the dipolar coupling strength in a three-spin molecule can be obtained by subtracting the two-spin and one-spin signals from the trimer's EPR spectrum, and finally the dipolar coupling strength in a four-spin molecule can be obtained by subtracting the one-, two- and three-spin signals from the tetramer's spectrum. In this way, tetramer **5** provides a scaffold for thoroughly characterizing qubit–qubit interactions in a multi-qubit system, from one high spin fraction reaction.

Rotation of N@C_60_ units around the common axis of the sp^3^-hybridized O–C and C–C bonds in each arm is likely to cause a variation in spin-to-spin distance of around 11 Å for adjacent units and 1 Å for mutually opposite units. While the variation in dipolar coupling strength due to such rotation would be negligible for mutually opposite units, a variation of around 4 MHz would be expected for adjacent units (see the electronic supplementary material for simulation details). Although distance variation would be averaged out, rotation of the N@C_60_ units is likely to somewhat reduce the accuracy of dipolar coupling strength measurements.

## 3. Conclusion

We have synthesized and characterized an N@C_60_-containing covalent array of four fullerene units, the largest such array reported to date. This demonstrates N@C_60_'s ability to form covalently linked arrays and provides a molecule which could be instrumental in characterizing qubit–qubit interactions in the scale-up of this qubit system. Such characterization would provide useful information for a future controlled entanglement experiment; however, this will require a high spin purity N@C_60_ sample. Work is currently being undertaken within our laboratory to achieve this.

## Supplementary Material

Experimental data

## Data accessibility

All data used in this research are included in the figures and tables in the main article and in the electronic supplementary material.
