# Supplementary material for: A porphyrin-centred fullerene tetramer containing an N@C60 substituent
Source: R Soc Open Sci. 2018 Jul 18;5(7):180338. doi: 10.1098/rsos.180338 (PMC6083688; doi:10.1098/rsos.180338)
Supplement: Experimental data [file rsos180338supp1.docx]

Supplementary information for

A porphyrin-centred fullerene tetramer containing an N@C_60_ substituent

Harry Macpherson, Stuart Cornes, Shen Zhou and Kyriakos Porfyrakis*

Contents

[General Remarks 2](#_Toc515480240)

[Synthetic procedures and characterisation 3](#_Toc515480241)

[NMR Spectra 5](#_Toc515480242)

[Mass spectra 6](#_Toc515480243)

[Fragmentation in MALDI 6](#_Toc515480244)

[Variation in spin-to-spin distance 9](#_Toc515480245)

[d_a1_ (27 Ångström) 11](#_Toc515480246)

[Program input for simulation (comments in green): 11](#_Toc515480247)

[d_a2_ (21 Ångström) 12](#_Toc515480248)

[Program input for simulation: 12](#_Toc515480249)

[d_a3_ (33 Ångström) 13](#_Toc515480250)

[Program input for simulation: 13](#_Toc515480251)

[d_o1_ (33 Ångström) 14](#_Toc515480252)

[Program input for simulation: 14](#_Toc515480253)

[References 15](#_Toc515480254)

# General Remarks

Unless otherwise stated, all solvents were used as purchased. Solvents were degassed with argon or nitrogen. Chromatography was undertaken using silica gel of particle size 40-63 μm. The N@C_60_ sample used to synthesise the final N@C_60_ tetramer was prepared using an optimised ion implantation procedure and was purified from approx. 100 ppm to 1500 ppm by recycling HPLC. The N@C_60_ fraction of the N@C_60_/C_60_ mixtures was determined using a combination of TEMPO ((2,2,6,6-tetramethylpiperidin-1-yl)oxidanyl) calibrated cw-EPR spectroscopy and C_60_ calibrated UV-Vis spectroscopy.

NMR spectra were recorded using Bruker AVIII400 and Bruker AVII 500 spectrometers. MALDI mass spectra were recorded on a Bruker Microflex LT spectrometer. X-Band cw-EPR measurements were performed on Magnettech Miniscope MS200 and Bruker EMX spectrometers. Simulations of cw-EPR spectra were performed using the EasySpin software package.

# Synthetic procedures and characterisation

Tetra-aldehyde **3**:

To a suspension of 4,4′,4′′,4′′′-(Porphine-5,10,15,20-tetrayl)tetrakis(benzoic acid) **1** (25 mg, 0.032 mmol) in dry CH_2_Cl_2_ was added 4-hydroxybenzaldehyde (31 mg, 0.254 mmol), EDC.HCl (49 mg, 0.256 mmol) and 4-dimethylaminopyridine (15 mg, 0.123 mmol). The mixture was left to stir at room temperature for 24 h after which purification was undertaken by column chromatography (SiO_2_, CH_2_Cl_2_/MeOH 1%) followed by recrystallisation from CH_2_Cl_2_, affording **3** as a purple solid (37 mg, 0.030 mmol, 20%). ^1^H NMR (400 MHz, Chloroform-*d*) δ 10.13 (s, 4H), 8.90 (d, *J* = 7.9 Hz, 8H), 8.81 (d, *J* = 8.0 Hz, 8H), 8.68 (s, 8H), 8.12 (d, *J* = 8.2 Hz, 8H), 7.64 (d, *J* = 8.2 Hz, 8H). MALDI-MS (negative): m/z: 1207.679 [M – H]- (C_76_H_46_N_4_O_12_ calc. 1206.31).

C_60_ tetramer **5**:

A suspension of tetra-aldehyde **3** (4.7 mg, 3.9 μmol), C_60_ (16.8 mg, 23 μmol) and *N*-(4-(hexyloxy)benzyl)glycine (20.7 mg, 78 μmol) in *o*-DCB (6 mL)was sonicated until all reagents were fully dissolved (~30 min). The mixture was then heated at 100^o^C for 2 h after which it was cooled to room temperature. The crude material was then purified by column chromatography (SiO_2_, *o*-DCB to *o*-DCB/EtOAc 1%) to afford tetramer **5** as a brown solid (5.7 mg, 1.2 μmol, 30%). ^1^H NMR (500 MHz, *d*_4_-*o*-DCB) δ 8.90 (s, 8H), 8.61 (d, *J* = 8.0 Hz, 8H), 8.40 (d, *J* = 8.2 Hz, 8H), 8.18 (d, *J* = 8.2 Hz, 8H), 7.64 (m, 16H), 7.05 (m, 16H), 5.27 (s, 4H), 4.92 (d, *J* = 9.5 Hz, 4H), 4.55 (d, *J* = 13.8 Hz, 4H), 4.19 (d, *J* = 10.7 Hz, 4H), 3.98 (m, 8H), 3.70 (d, *J* = 13.3 Hz, 4H), 1.80 – 1.74 (m, 8H), 1.46 (m, 8H), 1.32 (m, 16H), 1.31 (s, 2H), 0.87 (m, 12H). MALDI-MS (negative): m/z: 4903.517 [M – H]- (C_372_H_130­_N_8_O_12_) calc. 4902.99.

N@C_60_/C_60_ tetramer **5**:

A suspension of tetra-aldehyde **3** (3.8 mg, 3.1 μmol), N@C_60_/C_60_ (0.15% purity, 13.7 mg, 19 μmol) and *N*-(4-(hexyloxy)benzyl)glycine (10.1 mg, 38 μmol) in *o-*DCB (4 mL) was sonicated until all reagents were fully dissolved (~30 min). The mixture was then heated at 100^o^C for 1 h after which it was cooled to room temperature. The crude material was then purified by column chromatography (SiO_2_, *o*-DCB to *o*-DCB/EtOAc 1%). The EtOAc was removed from the fractions containing tetramer **5** by vacuum and the resulting *o*-DCB solution was used for the EPR measurements discussed in the main article. Removal of the *o*-DCB to give a solid sample of **5** led to complete loss of spin signal, and hence an accurate yield for the reaction could not be calculated.

# NMR Spectra

**Figure S1:** ^1^H NMR of tetra-aldehyde **3** in CDCl_3_ at 298 K (400 MHz).

**Figure S2**: ^1^H NMR of tetramer **5** in *d*_4_-*o*-DCB at 373 K (500 MHz).

# Mass spectra

## Fragmentation in MALDI

The MALDI spectra gathered of the final Prato reaction mixture as well as of isolated tetramer contain fragments. Most notably, the spectrum of the isolated tetramer shows a strong peak corresponding to a fragment formed by the loss of one fullerene, leaving a trimer containing an ylide group where the fullerene has dissociated. Dissociation of fulleropyrrolidines into the corresponding fullerenes and ylides in MALDI has been documented previously(1)(2) and is rationalised by a laser activated retro-cycloaddition reaction which produces pristine C_60_ and an azomethine ylide species. The ylide would be expected to be very reactive and so it is very unlikely that they are stable side products.

**Figure S3:** Mass spectrum of the C_60_ tetramer reaction mixture after 5 minutes of heating. Monomer calculated m/z 2130.48, dimer calculated m/z 3054.65 (DCTB matrix, negative mode).

**Figure S4:** Mass spectrum of the C_60_ tetramer reaction mixture after 15 minutes of heating. Dimer calculated m/z 3054.65, trimer calculated m/z 3977.82, tetramer calculated m/z 4902.99 (DCTB matrix, negative mode).

The mass spectrum at 15 minutes shows an additional peak at m/z 4183.911, which corresponds to an ylide shown in figure **S5**, which is most likely a fragment generated during ionisation. Another peak at an m/z ratio of 3260.3 corresponds to a trimer fragment in which one fullerene has been cleaved to leave an ylide group (calculated m/z 3259.84). The peaks at m/z 3324.58 and 3529.46 are unexplained, and may be formed due to reaction with the matrix.

**Figure S5:** Fragment of tetramer seen at m/z 4183.911 with a calculated m/z of 4184.01 (left) and fragment of the trimer seen at m/z 3460.02 with a calculated m/z of 3259.84 (right).

**Figure S6:** Mass spectrum at 60 minutes showing no dimer peak. Peaks at m/z 4183.868 corresponds to the ylide fragment and the peak at 4903.517 corresponds to tetramer **5** (DCTB matrix, negative mode).


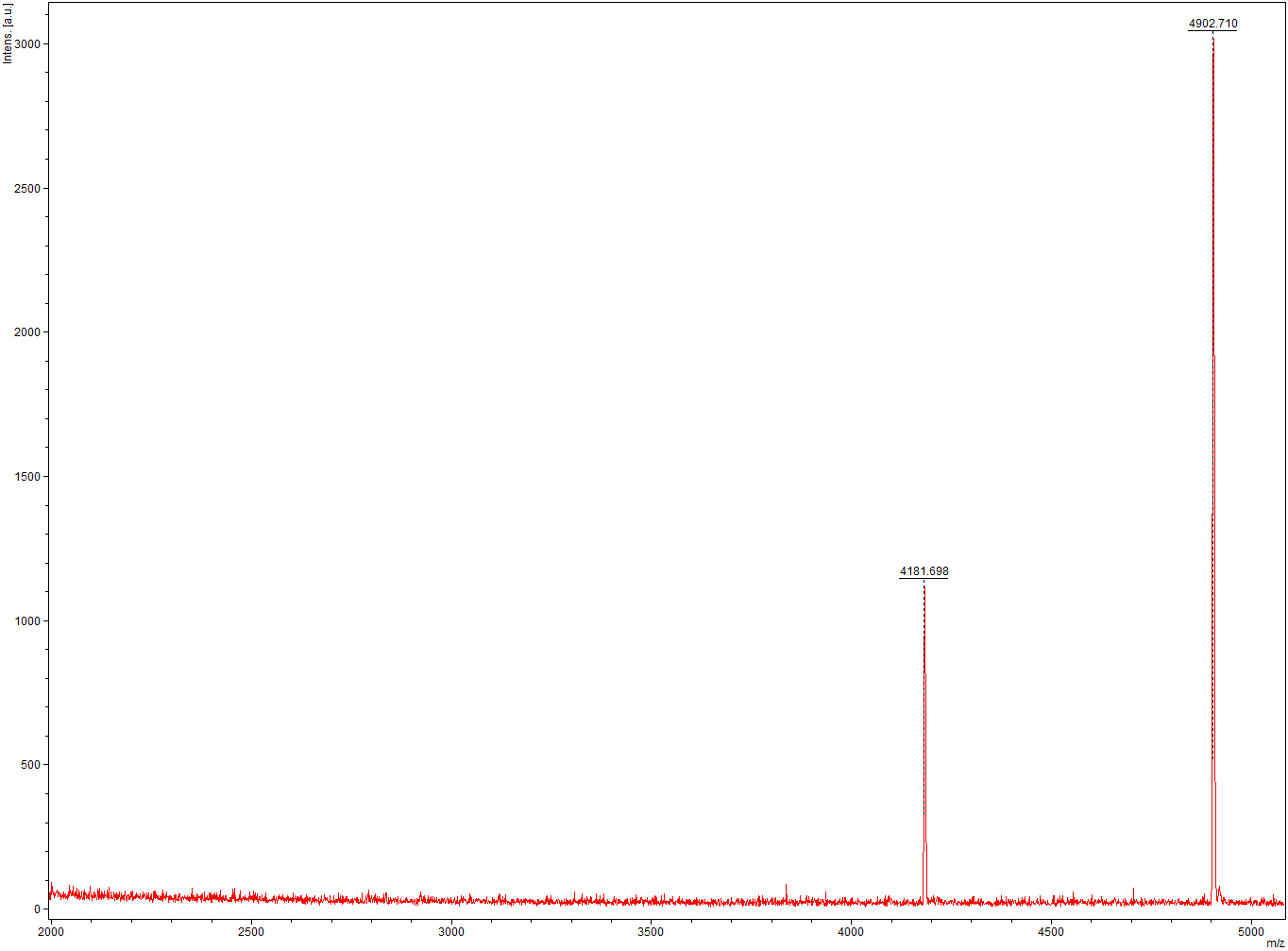


**Figure S7:** mass spectrum of isolated tetramer showing an ylide fragment peak and tetramer peak (DCTB matrix, negative mode).

# Variation in spin-to-spin distance

Since rotation of N@C_60_ units around the mutual axis of the sp^3^ hybridised O-C and C-C bonds of the tetramer’s arms is possible, X-Band cw-EPR spectra were simulated for different points in the bond rotation. The EasySpin line fitting tool was used with the program inputs as detailed below to generate spectra for different spin-to-spin distances depending on bond rotation (as shown in figure S8 and S9).

The simulated spectra and dipolar coupling strengths denoted “d_a_­_1_”, “d_a2_” etc., correspond to the interfullerene distances labelled likewise in figures S8 and S9.


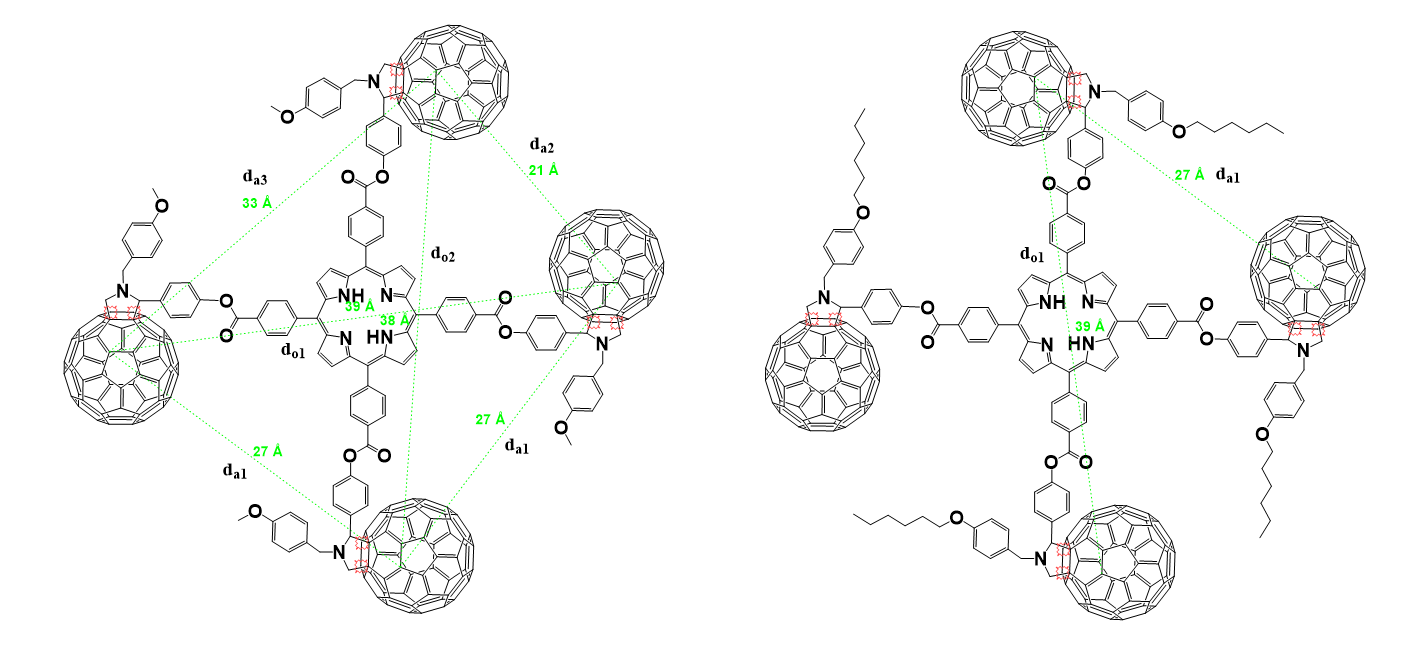


**Figure S8:** Interfullerene distances when one fullerene unit is rotated anti-clockwise.


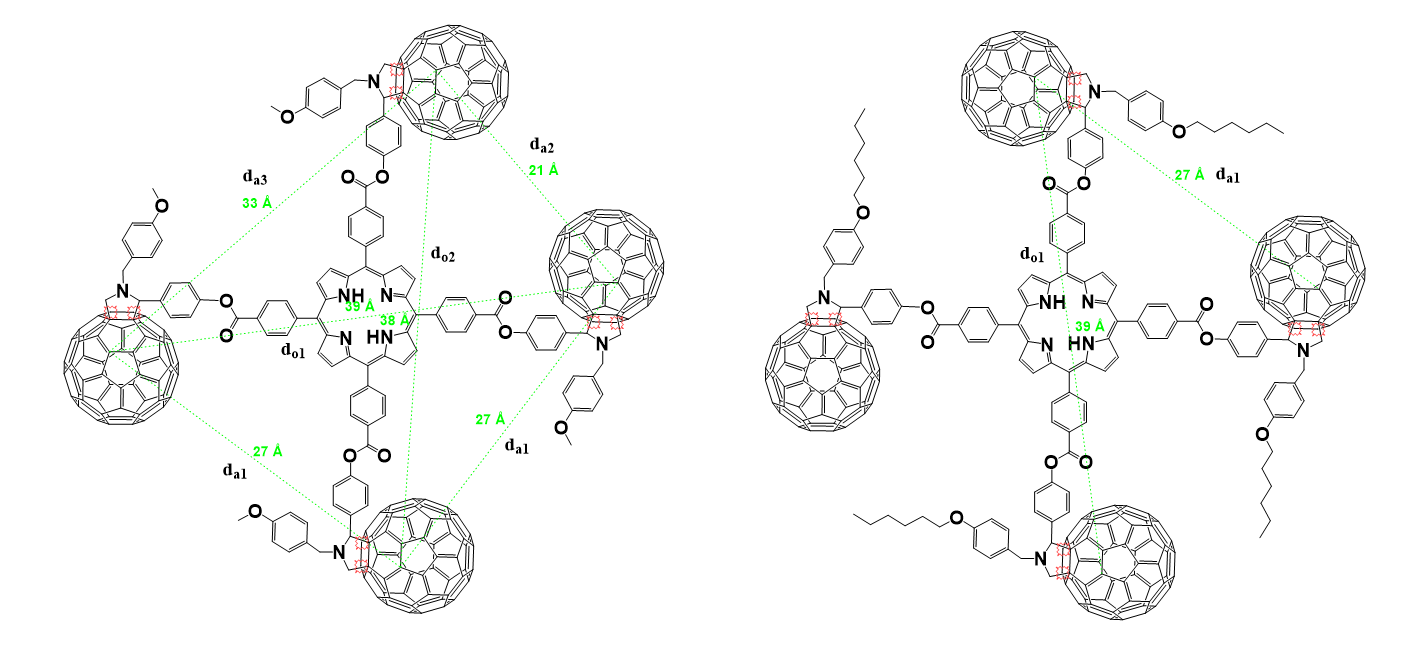


**Figure S9:** Interfullerene distances when all fullerenes are rotated clockwise.

## d_a1_ (27 Ångström)


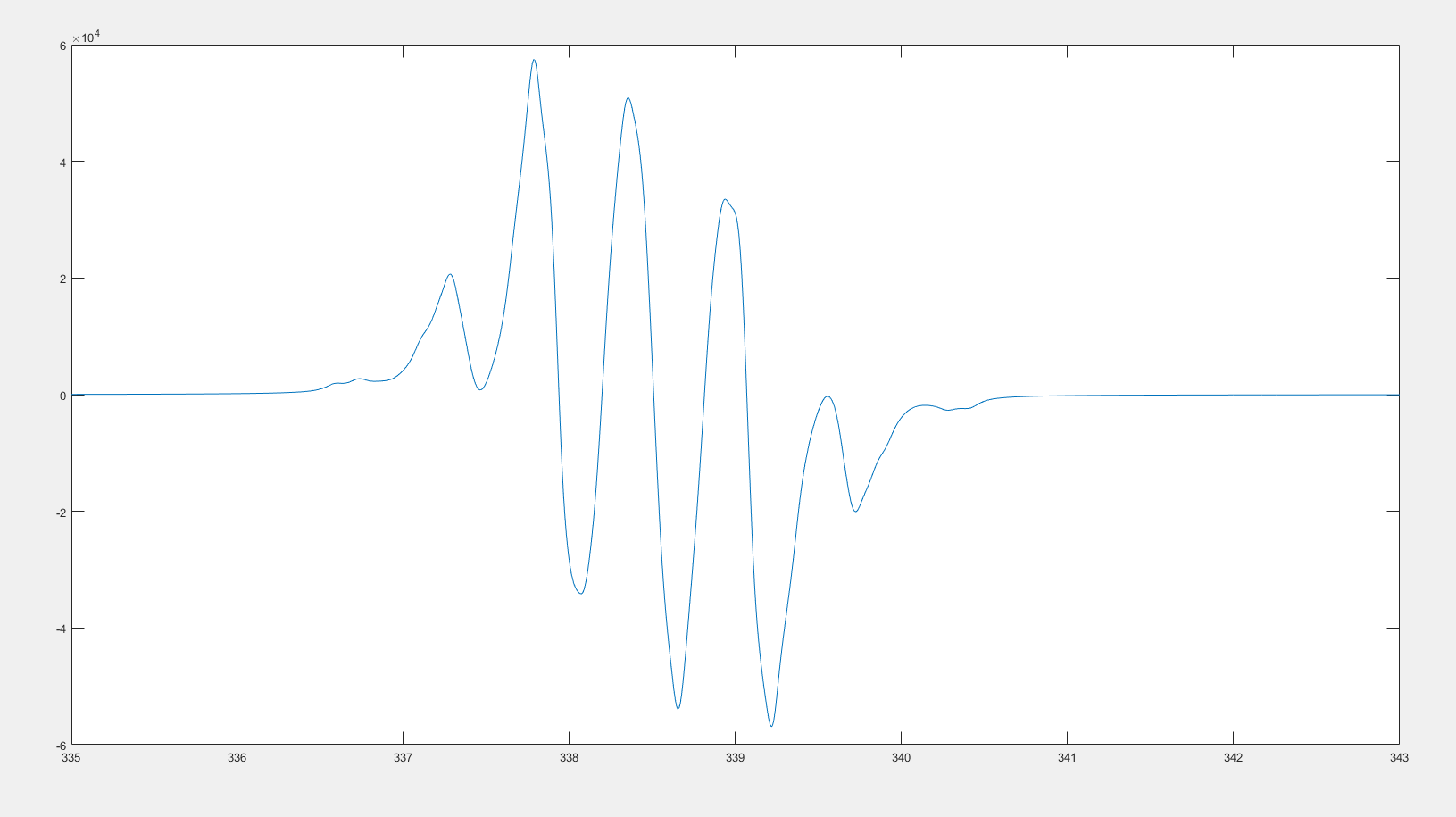


**Figure S10:** Simulated x-band cw-EPR spectrum for interfullerene distance d_a1_. The x-axis unit is milliTesla (mT).

The simulated dipolar coupling strength between two N@C_60_ units at 27 Å is 2.64 MHz

### Program input for simulation (comments in green):

clear, clc;
Sys1.g = [2.00514;2.00514]; %g factor; isotropic
Sys1.S = [3/2 3/2]; %Spin quantum number
Sys1.Nucs = 'N, N'; %nucleus
Sys1.A = [15.86 0;0 15.86]; %hyperfine coupling constant; isotropic; Unit: MHz
Coupling=2.64*[1,1,-2]; %2.7nm spin-to-spin distance
P1=[0,pi/180*45,0];%clockwise
P2=[0,pi/180*45,0];%clockwise
R1=erot(P1);
R2=erot(P2);
d=17.71;
e=0.63;
D=diag([-d/3+e ,-d/3-e ,2*d/3]);
Sys1.D = [R1*D*R1';R2*D*R2']; %zero-field splitting constant; isotropic; Unit: MHz
Sys1.ee=diag(Coupling);
Sys1.lwpp = [0.0 0.08]; %positive peak to negative peak distance; Unit: mT
Exp.mwFreq = 9.5; %Unit: GHz
Exp.Range = [335 343]; %Unit: mT
[x,y]=pepper(Sys1,Exp);
plot(x,y);

## d_a2_ (21 Ångström)


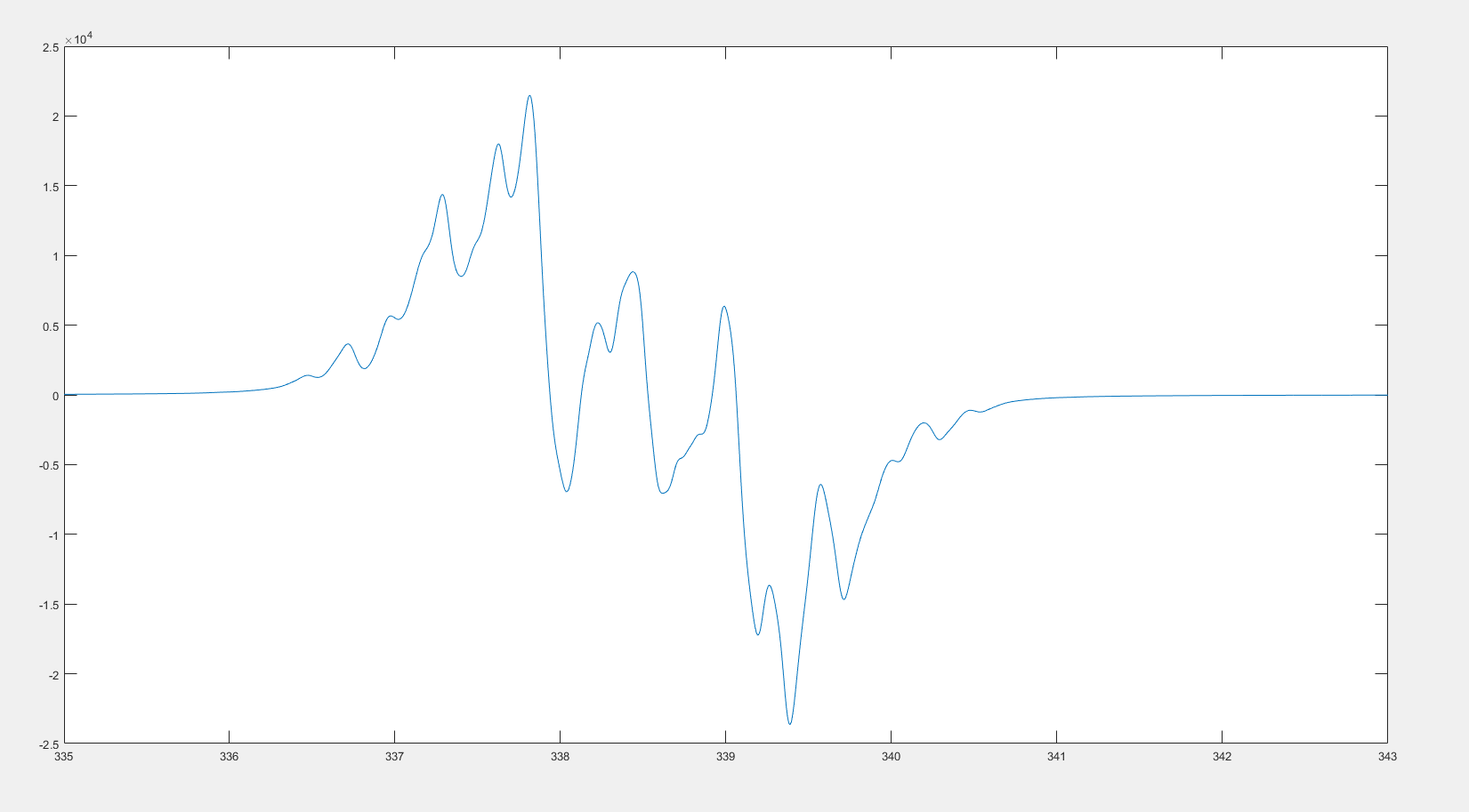


**Figure S11:** Simulated x-band cw-EPR spectrum for interfullerene distance d_a2_. The x-axis unit is milliTesla (mT).

The simulated dipolar coupling strength between two N@C_60_ units at 21 Å is 5.61 MHz.

### Program input for simulation:

clear, clc;
Sys1.g = [2.00514;2.00514];
Sys1.S = [3/2 3/2];
Sys1.Nucs = 'N, N';
Sys1.A = [15.86 0;0 15.86];
Coupling=5.61*[1,1,-2];
P1=[0,pi/180*45,0];
P2=[0,pi/180*135,0];
R1=erot(P1);
R2=erot(P2);
d=17.71;
e=0.63;
D=diag([-d/3+e ,-d/3-e ,2*d/3]);
Sys1.D = [R1*D*R1';R2*D*R2']; Sys1.ee=diag(Coupling);
Sys1.lwpp = [0.0 0.08];
Exp.mwFreq = 9.5;
Exp.Range = [335 343];
[x,y]=pepper(Sys1,Exp);
plot(x,y);

## d_a3_ (33 Ångström)


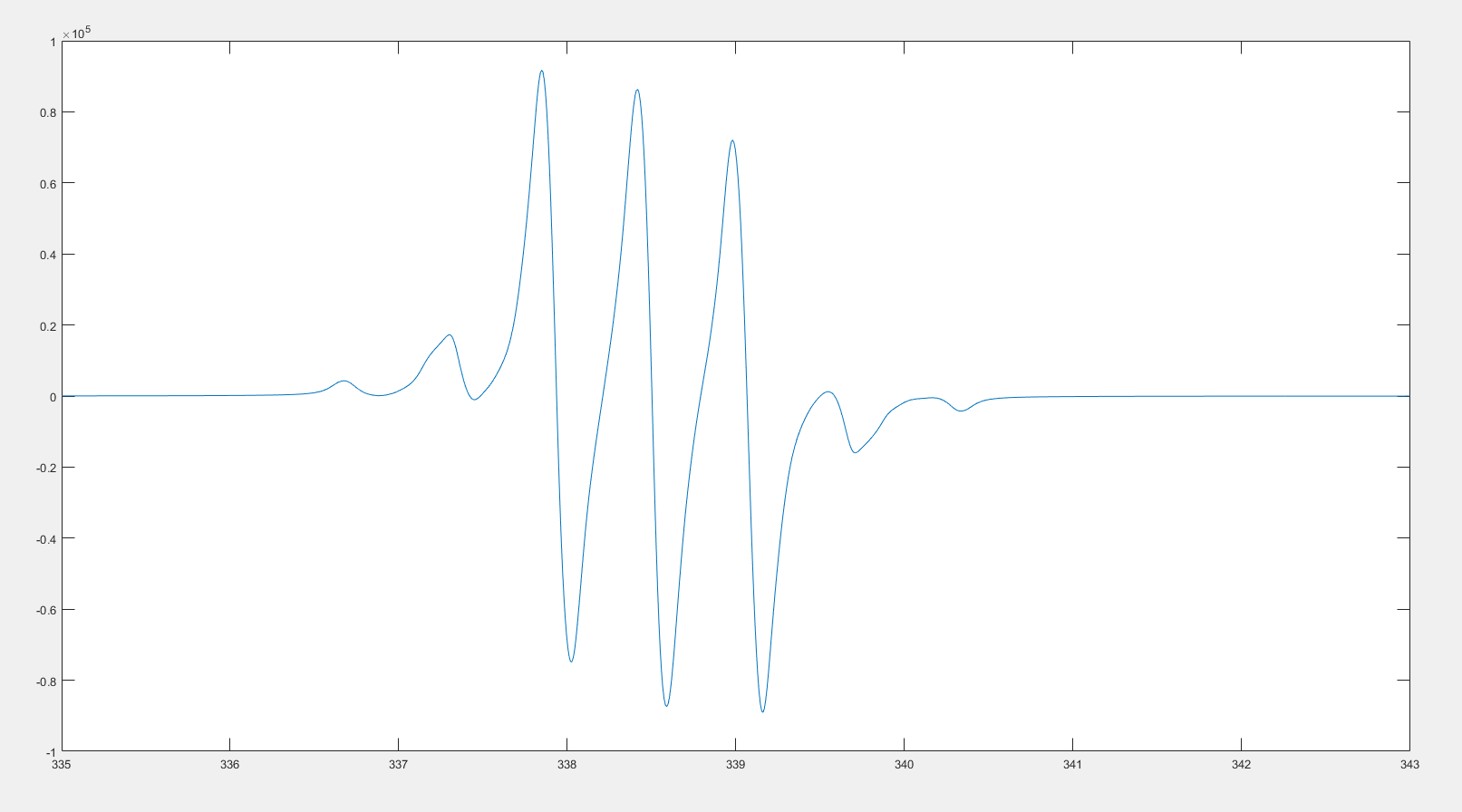


**Figure S12:** Simulated x-band cw-EPR spectrum for interfullerene distance d_a3_. The x-axis unit is milliTesla (mT).

The simulated dipolar coupling strength between two N@C_60_ units at 33 Å is 1.44 MHz.

### Program input for simulation:

clear, clc;
Sys1.g = [2.00514;2.00514];
Sys1.S = [3/2 3/2];
Sys1.Nucs = 'N, N';
Sys1.A = [15.86 0;0 15.86];
Coupling=1.44*[1,1,-2];
P1=[0,pi/180*45,0];
P2=[0,pi/180*135,0];
R1=erot(P1);
R2=erot(P2);
d=17.71;
e=0.63;
D=diag([-d/3+e ,-d/3-e ,2*d/3]);
Sys1.D = [R1*D*R1';R2*D*R2'];
Sys1.ee=diag(Coupling);
Sys1.lwpp = [0.0 0.08];
Exp.mwFreq = 9.5;
Exp.Range = [335 343];
[x,y]=pepper(Sys1,Exp);
plot(x,y);

## d_o1_ (33 Ångström)


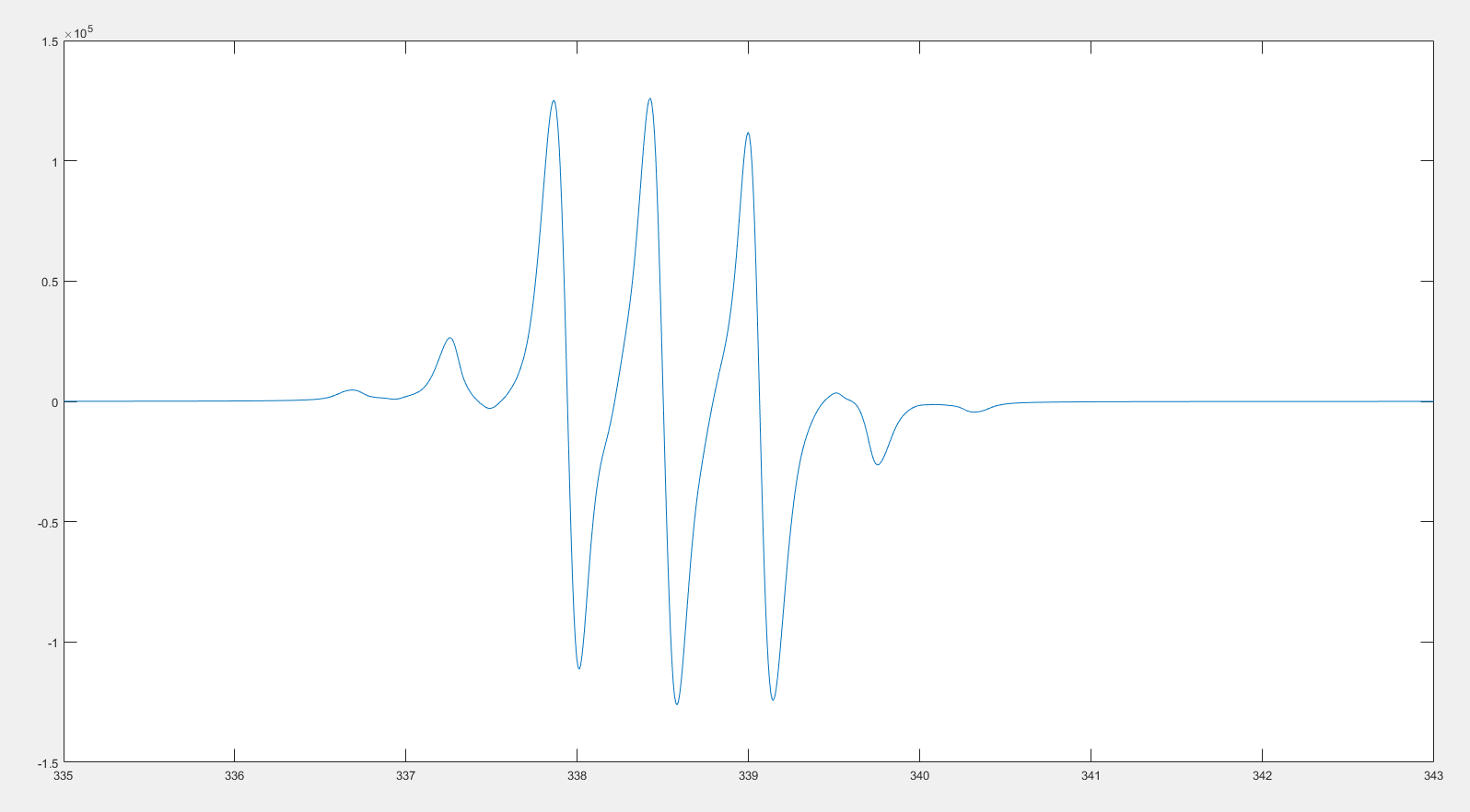


**Figure S13:** Simulated x-band cw-EPR spectrum for interfullerene distance d_o1_. The x-axis unit is milliTesla (mT).

The simulated dipolar coupling strength between two N@C_60_ units at 39 Å is 0.88 MHz.

### Program input for simulation:

clear, clc;

Sys1.g = [2.00514;2.00514];

Sys1.S = [3/2 3/2];

Sys1.Nucs = 'N, N';

Sys1.A = [15.86 0;0 15.86];

Coupling=0.88*[1,1,-2];

P1=[0,pi/180*85,0];

P2=[0,pi/180*-95,0];

R1=erot(P1);

R2=erot(P2);

d=17.71;

e=0.63;

D=diag([-d/3+e ,-d/3-e ,2*d/3]);

Sys1.D = [R1*D*R1';R2*D*R2'];

Sys1.ee=diag(Coupling);

Sys1.lwpp = [0.0 0.08];

Exp.mwFreq = 9.5;

Exp.Range = [335 343];

[x,y]=pepper(Sys1,Exp);

plot(x,y);

# References

1. Bottari G, Dammann C, Torres T, Drewello T. Laser-induced azomethine ylide formation and its covalent entrapment by fulleropyrrolidine derivatives during MALDI analysis. J Am Soc Mass Spectrom. 2013;24(9):1413–9.

2. Delgado JL, Osuna S, Bouit PA, Martínez-Alvarez R, Espíldora E, Solá M, et al. Competitive retro-cycloaddition reaction in fullerene dimers connected through pyrrolidinopyrazolino rings. J Org Chem. 2009;74(21):8174–80.
